# Early Diagnosis and Surgical Management of Tropical Chronic Pancreatitis in a Tertiary Care Rural Hospital: A Case Report

**DOI:** 10.7759/cureus.49826

**Published:** 2023-12-02

**Authors:** Yashraj Jain, Rajesh G Gattani, Raju K Shinde, Swati G Deshpande

**Affiliations:** 1 General Surgery, Jawaharlal Nehru Medical College, Datta Meghe Institute of Higher Education and Research, Wardha, IND

**Keywords:** pancreatic diseases / surgery, partington-rochelle procedure, early diagnosis and treatment, lateral pancreatico jejunostomy, calcific pancreatitis, surgical management of pancreatitis, chronic calcific pancreatitis

## Abstract

Tropical chronic pancreatitis represents a variant of chronic calcific non-alcoholic pancreatitis, typically found in adolescents and young adults, predominantly in developing nations. This condition usually presents as a classic triad of recurrent and severe abdominal pain, diabetes, and steatorrhea. As the disease progresses and diabetes develops, it is called fibrocalculous pancreatic diabetes. A defining characteristic of this ailment is the presence of pancreatic calculi, leading to duct dilation. Key features of this condition include an early onset in youth, intraductal calculi, an aggressive disease course, and a heightened susceptibility to pancreatic cancer. Diagnostic tools such as ultrasound, CT, magnetic resonance cholangiopancreatography, and endoscopic retrograde cholangiopancreatography aid in identifying the disease. Timely diagnosis and treatment significantly reduce mortality and morbidity. Our patient, a young female, presented solely with recurrent episodes of abdominal pain resembling pancreatitis, along with a normal biological profile and an absence of readily apparent symptoms. She received a diagnosis of tropical chronic pancreatitis and underwent the Partington-Rochelle procedure for surgical decompression of the main pancreatic duct.

## Introduction

Chronic pancreatitis is a progressively inflammatory condition that leads to the deterioration of the endocrine and exocrine tissues within the pancreas, primarily due to the replacement of these tissues with fibrotic material [1]. In contrast, tropical pancreatitis is a specific form of chronic pancreatitis observed in tropical regions [2]. This variant predominantly affects young adults and juveniles. It is characterized by recurring epigastric pain, the presence of pancreatic calcifications, the development of diabetes, and a higher propensity to develop pancreatic malignancies [2]. Initially documented in Indonesia, most cases have since been reported in southern India [3].

The precise cause of tropical chronic pancreatitis remains unknown. Various factors, such as malnutrition, cassava consumption, a diet deficient in protein, and autoimmune and genetic factors, have been proposed as potential causes. However, no definitive studies have substantiated these associations [4]. The presence of a SPINK1 mutation has been linked to the occurrence of tropical calcific pancreatitis [4].

There is no universally accepted gold standard for diagnosing chronic calcific pancreatitis [5]. CT is commonly utilized and considered a dependable method for evaluating the morphology of the pancreas. Magnetic resonance cholangiopancreatography, more sensitive than CT, is employed when CT scans yield inconclusive results [5]. It offers exceptional visualization of the pancreatic duct. While endoscopic ultrasound is even more sensitive, its invasive nature means it is typically reserved for cases that are challenging to diagnose [5]. Endoscopic retrograde cholangiopancreatography is a highly effective test for demonstrating dilation or stricture of the pancreatic duct and its branches. It can sometimes serve diagnostic and therapeutic purposes [5]. Pancreatic function tests are also conducted to assess exocrine function [5].

## Case presentation

A 22-year-old woman, married and with a ten-month-old child, hails from a rural background. She presented with a progressively worsening chronic upper abdominal pain that had troubled her for eight years. The abdominal pain, which radiated to her back, occurred intermittently. The frequency of these episodes, the duration of each episode, and the factors related to the onset of pain were not explicitly detailed in her initial presentation. The pain had been managed conservatively at a rural healthcare center. Apart from this persistent pain, she did not report any other significant complaints.

**Figure 1:**
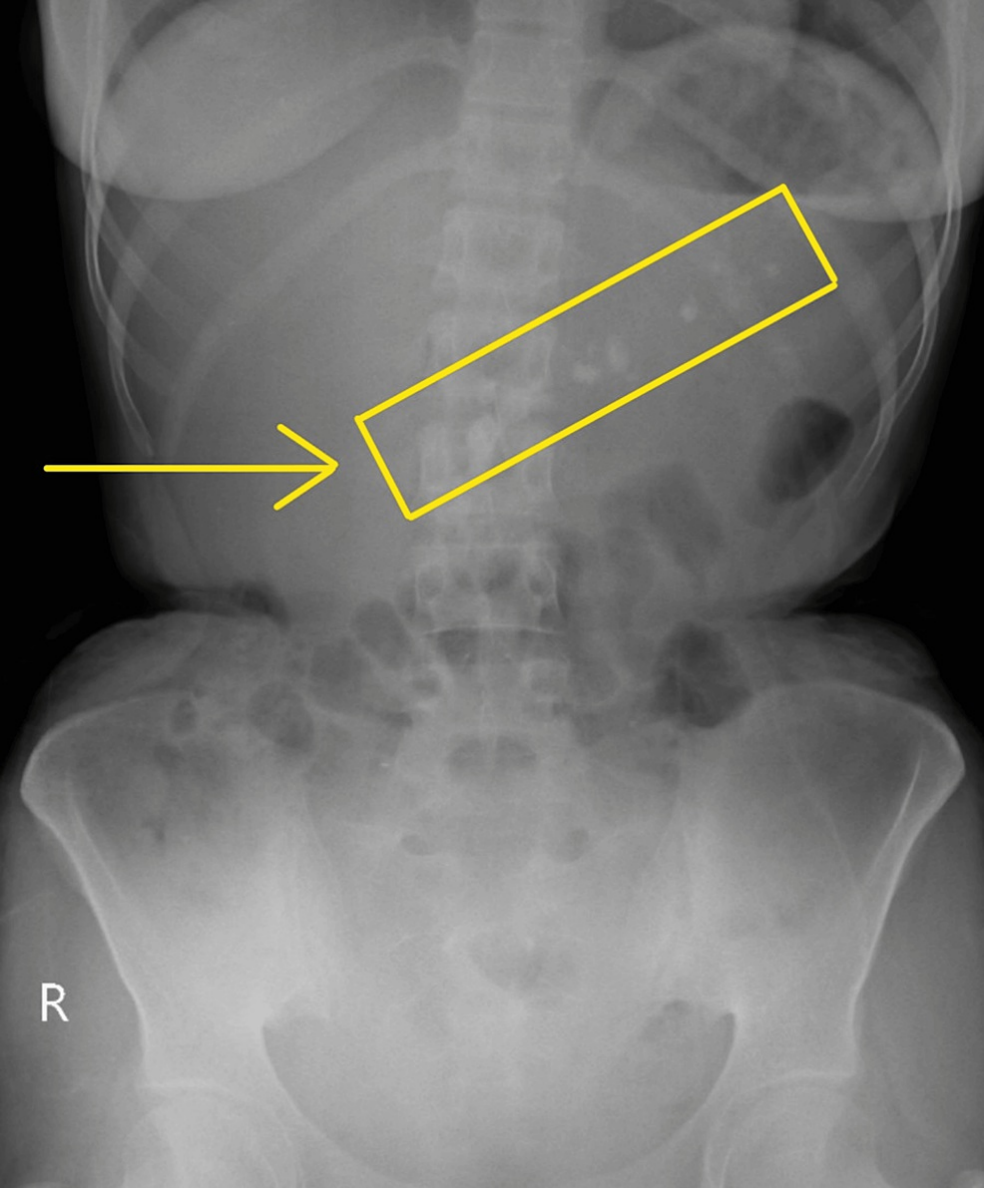
X-ray abdomen showing multiple calculi

Coming from a low-income family, the patient had no history of cassava consumption, jaundice, alcohol use, or abdominal trauma. During the examination, the patient was found stable, with a low body mass index of 14, displaying no signs of jaundice or pallor. Her abdomen was soft and non-tender, with no guarding or rigidity, and she had normal bowel sounds. The details of her laboratory investigations are given in Table 1. X-ray of the abdomen showed multiple calculi (Figure 1).

**Table 1:** Laboratory investigations Hb: Haemoglobin; TLC: Total leukocyte count; SGOT: Serum glutamic oxaloacetic transaminase; SGPT: Serum glutamate pyruvic transaminase; ALP: Alkaline phosphatase; RBS: Random blood sugar

Name of the investigation	Value
Hb %(gm/dl)	10.5
TLC (mm^3^)	8800
Platelet (lakhs/mm^3^)	2.99
Total bilirubin (mg/dL)	0.7
Conjugated bilirubin (mg/dL)	0.3
SGOT (IU/L)	20
SGPT (IU/L)	25
ALP (IU/L)	100
Urea (mg/dl)	35
Creatinine (mg/dl)	0.9
Sodium (mEq/L)	135
Potassium (mEq/L)	4.3
Calcium (mg%)	9.8
Chloride	103
Amylase (U/L)	49
Lipase (U/L)	14
RBS (mg/dl)	81

**Figure 2:**
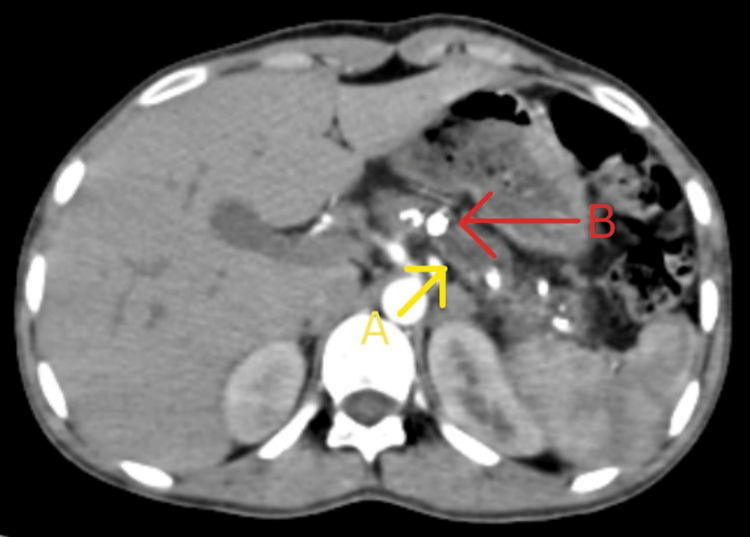
CT of the abdomen showing dilated main pancreatic duct with multiple calculi (axial view) A: Dilated main pancreatic duct; B: Intraductal calculi

**Figure 3:**
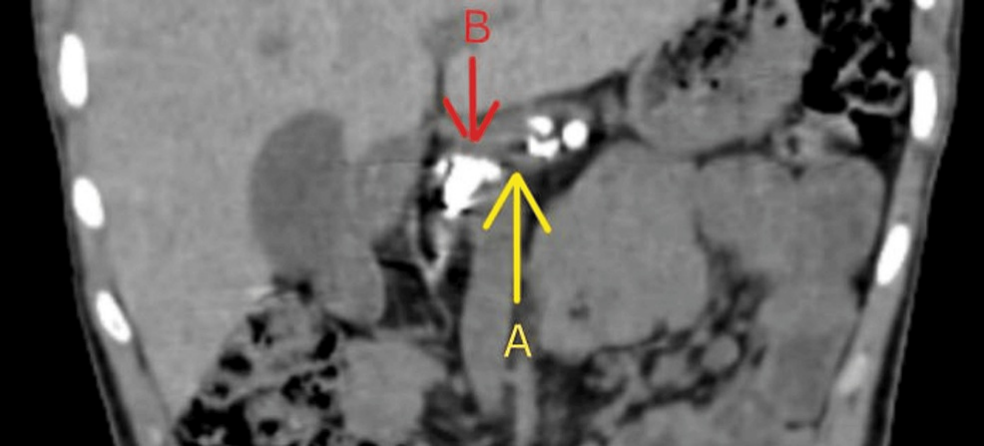
CT of the abdomen showing dilated main pancreatic duct with multiple calculi (coronal view) A: Dilated main pancreatic duct; B: Intraductal calculi

The abdominal CT revealed findings consistent with chronic pancreatitis, including a small and dilated main pancreatic duct and multiple intraductal calculi. Notably, the largest of these calculi measured 1.6 cm in diameter (Figures 2, 3).

**Figure 4:**
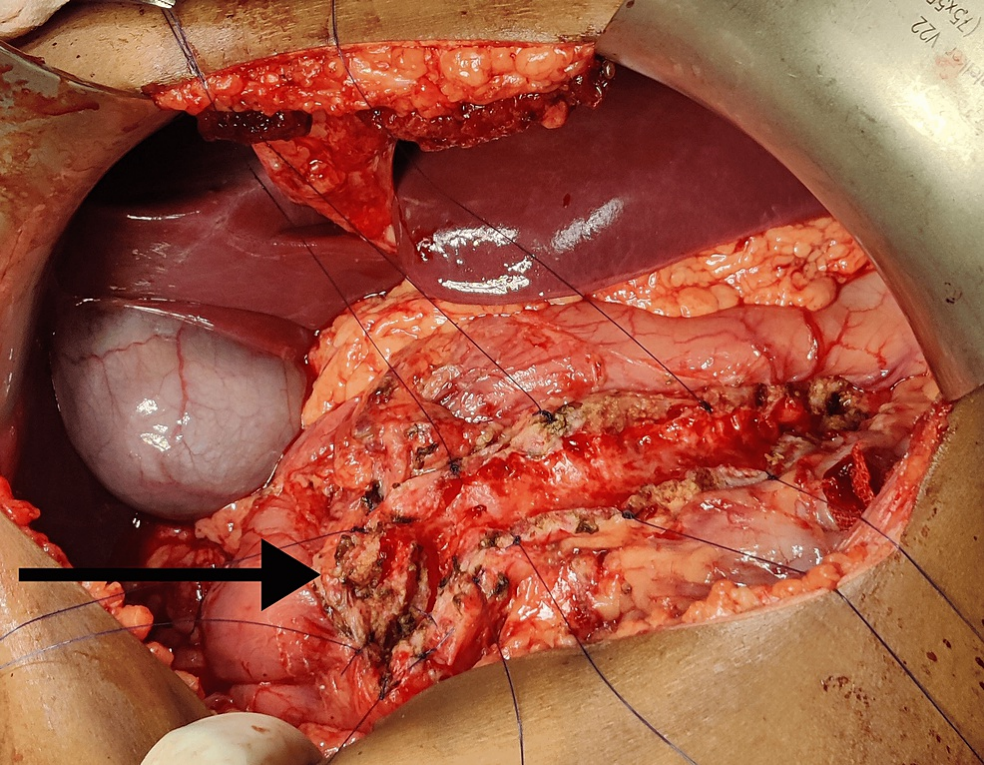
Laid-open main pancreatic duct

**Figure 5:**
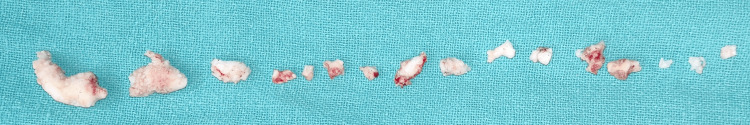
Evacuated calculi from pancreatic duct

There was no evidence of dilatation in the common bile duct, and the liver exhibited a normal appearance. Subsequently, a Partington-Rochelle procedure was conducted for the patient. This surgical intervention involved making a Chevron incision, opening the gastrocolic ligament and separating the stomach from the anterior section of the pancreas. The surface of the pancreas was carefully examined by palpation to locate the pancreatic duct. With the aid of a syringe, the duct's position was precisely determined. The duct was then incised lengthwise, and the pancreatic duct was laid open (Figure 4). This procedure allowed for the evacuation of calculi from the main pancreatic duct, as illustrated in Figure 5.

A Roux limb was fashioned approximately 20 cm from the ligament of Treitz. A window was carefully created in the transverse mesocolon, situated to the right of the middle colic artery, facilitating the maneuvering of the distal Roux limb into the supra-colic compartment. Subsequently, a pancreaticojejunostomy was performed, and to maintain bowel continuity, an end-to-side jejunojejunostomy was established. The patient's hospital stay was extended to seven days without any noteworthy incidents. Plans for further monitoring include regular follow-up appointments, imaging studies as deemed necessary, and ongoing assessments of abdominal pain and blood sugar levels. At the one-month follow-up, the patient reported the absence of abdominal pain, and her blood sugar levels had returned to within the normal range. Continued monitoring will be essential to assess the long-term effectiveness of the surgical intervention and the patient's overall well-being.

## Discussion

The primary objective of surgical intervention in cases of tropical pancreatitis is to alleviate chronic, unyielding pain, a debilitating concern for these patients [6]. Early diagnosis, coupled with appropriate treatment, plays a pivotal role in reducing complications associated with chronic pancreatitis [7]. In our case, the patient sought medical attention before experiencing endocrine deficiencies. Given the patient's youth and her responsibilities as a mother to a young child, prompt treatment was of paramount importance. The classic clinical presentation of tropical calcific pancreatitis typically includes abdominal pain, diabetes, and steatorrhea [8]. However, in our patient's case, only abdominal pain had manifested, while the other symptoms were notably absent, suggesting an early stage of the disease.

Several potential causes have been proposed for tropical calcific pancreatitis, including cassava consumption, undernutrition, and the SPINK1 mutation. However, none of these factors has been definitively established as the sole etiology [9]. In tropical calcific pancreatitis, the calculi are characterized by their large size and presence in the main pancreatic duct, distinguishing them from the small stones seen in small ducts, which are typical in hereditary and alcoholic pancreatitis [10].

This case stands out due to its early presentation and the rarity of tropical chronic pancreatitis. The Partington-Rochelle procedure was chosen to address the need for a longitudinal incision for evacuating multiple stones from the pancreatic duct. While a randomized control trial has indicated that this procedure offers an improved quality of life and effective pain relief [11], the specific indications, pros, and cons of the Partington-Rochelle procedure warrant further elaboration.

Given the ambiguous etiology and the risk of recurrence in this subtype of pancreatitis, it is crucial to explore the duration of the procedure's effectiveness and the recommended follow-up duration for patients. Additionally, in our case, the patient presented with abdominal pain but without diabetes or steatorrhea, prompting consideration of the variable clinical manifestations of tropical chronic pancreatitis.

## Conclusions

Tropical chronic pancreatitis, although uncommon, poses a considerable threat to patient well-being when not promptly addressed. Effective management yields positive results and enhances the overall quality of life for affected individuals. This report adds to the body of knowledge on tropical chronic pancreatitis, offering valuable insights into successful management approaches that can lead to favorable outcomes.
